# Lgr5^+^ cells are required and dynamically participate in olfactory epithelium regeneration: a revisiting shows Lgr5 expression in multiple cell lineages

**DOI:** 10.7150/thno.60636

**Published:** 2022-07-18

**Authors:** Wenwen Ren, Zhenjie Ma, Li Wang, Xiaoyu Feng, Hongmeng Yu, Yiqun Yu

**Affiliations:** 1 Ear, Nose & Throat Institute, Department of Otolaryngology, Eye, Ear, Nose & Throat Hospital, Fudan University, Shanghai 200031, China.; 2 Department of Otolaryngology, the Second Affiliated Hospital of the Naval Military Medical University (Shanghai Changzheng Hospital), Shanghai, China.; 3 School of Life Sciences, Shanghai University, Shanghai 200444, China.; 4 Clinical and Research Center for Olfactory Disorders, Eye, Ear, Nose & Throat Hospital, Fudan University, Shanghai 200031, China.; 5 Research Units of New Technologies of Endoscopic Surgery in Skull Base Tumor, Chinese Academy of Medical Sciences, Beijing 100730, China.

**Keywords:** Lgr5, olfactory epithelium, regeneration, olfactory sensory neuron, globose basal cell, horizontal basal cell

## Abstract

Olfactory sensory neurons (OSNs) located in the olfactory epithelium (OE) detect thousands of volatile environmental odors to form the sense of smell. OSNs are generated from basal cells, which show the characteristics of progenitor/stem cells. In the mammalian OE, persistent neurogenesis occurs during lifetime, providing a unique model to study the tissue turnover and fate determination of stem cells.

**Methods:** Immunohistochemical analysis and RNAscope in situ hybridization indicated the localization of leucine-rich repeat-containing G-protein-coupled receptor 5 (Lgr5) in the intact and injured OE. Lineage tracing was conducted to analyze the dynamic role of Lgr5^+^ cells in the OE homeostasis and regeneration. We also used DTR-driven genetic depletion of Lgr5^+^ cells and lentivirus-mediated Lgr5 downregulation to demonstrate the essential role of Lgr5^+^ cells in the OE regeneration.

**Results:** We show that Lgr5 marks horizontal basal cells (HBCs) in the OE of adults but not newborns. We revisit the role of Lgr5^+^ cells in the OE homeostasis and regeneration, and find that Lgr5^+^ cells participate in the OE homeostasis from neonatal to one-month-old age, as well as in the OE regeneration post injury. During the OE regeneration, Lgr5 is transiently expressed in apical supporting cells, immature neurons, and mature sensory neurons. The Lgr5^+^ cells become or generate HBCs in the regenerated OE. DTR-driven cell depletion shows that Lgr5^+^ cells are not necessary in the adult OE homeostasis, but required in the recovery of OE from injury. Lgr5 down-regulation by lentiviral infection also demonstrates the essential role of Lgr5 expression in the OE regeneration.

**Conclusion:** Our study elucidates the role of Lgr5^+^ cells in the OE homeostasis and regeneration, potentially providing a candidate to cell-based therapy against olfactory dysfunction.

## Introduction

The olfactory epithelium (OE) is a pseudostratified epithelial structure mainly composed of apical sustentacular/supporting cells, mature olfactory sensory neurons (OSNs), immature OSNs, globose basal cells (GBCs) and horizontal basal cells (HBCs) 1. Adult neurogenesis constitutively occurs in the mammalian OE throughout life 2. During this process, GBCs and HBCs in the basal compartment function as stem/progenitor cells to generate OSNs and supporting cells 3-7. The GBCs show a strong proliferative capacity, with both transient amplifying cells committed to a neuronal lineage (GBCs that are ostensibly committed to making neurons) and immediate neuronal precursors (GBCs that make neurons) contained in this cellular subtype 8. Compared to active GBCs generating sensory neurons, HBCs are quiescently reserved and remain inactive in OE homeostasis 9, 10. After sensory neurons are selectively killed by olfactory bulbectomy, GBCs differentiate into OSNs while HBCs are still quiescent 11. By comparison, when the whole epithelium is wiped out with exposure to toxic reagents such as methimazole, HBCs are recruited and contribute to OE regeneration 12, while supporting cells can arise by direct fate conversion from olfactory HBCs without cell division 13. This demonstrates that HBCs may serve as a reserve stem cell pool. Olfactory bulbectomy (OBX) reprograms GBCs to multipotency, producing supporting cells and other non-neuronal lineages in the OE 14. Similarly, methimazole lesion also reprograms neuronally specified GBCs to generate supporting cells, microvilli cells and sensory neurons 14. This suggests a transition in basal cells from unipotent specified differentiation to multipotent state when the OE is subjected to injury. Besides, the ablation of supporting cells but not neuronal depletion in the OE is sufficient for the HBC activation 10, 15, while regulation of secreted protein from supporting cells controls neuronal regeneration in the injured OE 16. Thus, supporting cells may affect basal cell activation and subsequent OE regeneration.

Two types of basal cells in the OE express different markers. For heterogeneous GBCs, stem-like cells express Sox2^+^/Pax6^+^^17^ and transit-amplifying progenitors with a limited proliferative capacity express Mash1 18, while immediate precursor cells that make neurons directly express Neurog1 and NeuroD1^19^. Mitotically quiescent HBCs are homogeneous and express cytokeratin5 (Krt5), Krt14, ICAM-1 and the transcription factor p63 4, 20. Upon OE injury, a subtype of activated HBCs appears, with the expression of Krt6a, Krtdap and Sprr1a 21. This type of HBCs has a transient state and is unique to regeneration.

In recent years, leucine-rich repeat-containing G-protein-coupled receptor 5 (Lgr5), a member of the *Wnt* signaling pathway, which plays critical roles in embryonic development and adult cell genesis in various tissues 22, has been established as an adult stem cell marker in small intestine and colon 23, 24, stomach 25, 26, hair follicle 27, kidney 28, mammary gland 29, 30 and ovary 31, 32. Lgr5 also labels stem and progenitor cells in sensory organs such as eye 33, ear 33-35, and tongue 36. In nose, Lgr5 marks the GBCs located in the OE and functions in the recovery of injured OE 37-39. However, how Lgr5^+^ cells behave in the homeostasis and regeneration of OE is still not very clear. Understanding this may facilitate to decipher the mechanism underlying self-repair in the OE.

In this study, we revisited the role of Lgr5^+^ cells in homeostasis and regeneration of the OE. Through lineage tracing, we did not specify an active role of Lgr5^+^ cells in the adult OE homeostasis, while Lgr5^+^ cells participated in the regeneration. Lgr5^+^ cells were transiently present in multiple cell lineages including immature sensory neurons, mature neurons and supporting cells during the OE regeneration, and became or gave rise to HBCs in the fully regenerated OE, showing a dynamic role of Lgr5^+^ cells. Through genetic depletion, we confirmed that Lgr5^+^ cells were not necessary in the maintenance of normal OE, but were required in the regeneration of injured OE. Under OBX-induced OE injury, Lgr5^+^ cells displayed multipotent state, generating both sensory neurons and supporting cells. Collectively, this work elucidated the performance of Lgr5^+^ cells in the OE homeostasis and regeneration, implying an ideal candidate to the cell replacement-based therapy against olfactory dysfunction.

## Methods

### Animals

Wide type C57BL/6J mice were purchased from Shanghai Model Organisms. Genetically targeted heterozygous Lgr5-EGFP-IRES-Cre^ERT2^ mice (Stock number 008875; harboring a "knock-in" allele that abolishes Lgr5 gene function and expresses EGFP and Cre^ERT2^ fusion protein from the Lgr5 promoter/enhancer elements), Rosa26-floxed STOP-TdTomato mice (Stock number 007909; a cre reporter strain with a loxP-flanked STOP cassette prevents transcription of the downstream red fluorescent protein) and Rosa26-floxed STOP-Diphtheria Toxin Receptor (Rosa26-iDTR, Stock number 007900, the Cre-inducible expression of DTR render cells susceptible to ablation following diphtheria toxin administration) were purchased from the Jackson Laboratory. Both male and female mice were used in this study and the data were grouped together because no sex difference was evident. The procedures of animal handling and tissue harvesting were approved by the institutional animal care and use committee (Permit Number: 2009-0082).

### Methimazole injection

For the OE lesion, 2-3-month-old animals were intraperitoneally injected with methimazole (50 μg/g body weight, Sigma Aldrich) as previously reported 11. In the saline control, the animals were injected with the same amount of PBS.

### Lineage tracing

For genetic lineage tracing, Lgr5-EGFP-IRES-Cre^ERT2^ mice were crossed with Rosa26-TdTomato mice. To induce the expression of the reporter gene, a single dose or three consecutive doses (one dose per day) of tamoxifen (0.22 mg/g body weight, Sigma Aldrich) was injected intraperitoneally at the age of three-month-old. Mice were then sacrificed at different time points after tamoxifen induction. Sunflower oil (dissolvent to tamoxifen, purchased from Sigma Aldrich) was injected in control mice. For the tamoxifen induction in neonatal mice at P0, one dose of tamoxifen was injected into the newborns. For lineage tracing of Lgr5^+^ cells in the injured OE, methimazole was administrated at Day 1 followed by three doses of tamoxifen injection. The scheme for the experimental design was shown in Figure S4.

### Genetic ablation of Lgr5^+^ cells

For the genetic deletion of Lgr5^+^ cells, Lgr5-EGFP-IRES-Cre^ERT2^ mice were crossed with Rosa26-iDTR mice. Five doses of diphtheria toxin (Sigma Aldrich) at 10 ng/g body weight were injected by one-day interval between two injections. Tamoxifen (0.22 mg/g body weight) was injected on the day before each dose of diphtheria toxin. In the injured OE, three and two doses of combination of tamoxifen and diphtheria toxin were administrated before and after methimazole injection, respectively. Mice were then sacrificed at Day 17, 31, 60 post the first dose of tamoxifen injection without methimazole exposure, or at Day 3, 10, 17, 31 post methimazole-induced injury with tamoxifen injection. The experimental design was shown as Figures 4A and S5A.

### Unilateral olfactory bulbectomy and tamoxifen induction

Olfactory bulbectomy (OBX) was performed as described previously 40. Briefly, Lgr5-EGFP-IRES-Cre^ERT2^/Rosa26-TdTomato mice at three-month-old age were anesthetized and immobilized in a stereotactic mount. The frontal bone was exposed by a single incision on sterilized skin. A bone drill exposed the bulb on right hemisphere and the single olfactory bulb was ablated by suction. The ablation cavity was placed with sterile oxycel, and skin was sutured. The animals undergoing surgery were injected with antibiotics to avoid infection. Three doses of tamoxifen induction were performed three days prior to OBX, and animals were sacrificed at Day 10 and Day 31 post OBX (Figure 6A).

### Immunohistochemistry

Mice were deeply anesthetized by intraperitoneal injection of ketamine-xylazine (200 and 15 mg/kg body weight) before decapitation. The heads were fixed in 4% paraformaldehyde (Sigma Aldrich) overnight at 4 ºC, and infiltrated in a series of sucrose solutions before being embedded in OCT. The frozen tissues were cut into 20 μm coronal sections on a cryostat (Leica CM1950). After rinsing with PBS, the tissue sections were blocked for 1 h in 0.3% Triton X-100 in phosphate-buffered saline with 5% bovine serum albumin, and then incubated at 4 °C with the primary antibodies overnight. The primary antibodies included goat anti-Sox2 (1:100, Santa Cruz, sc-17320; and 1: 500, R&D, AF2018), rabbit anti-Sox2 (1:100, Proteintech, 11064-1-AP), goat anti-NeuroD1 (1:500, Santa Cruz, sc-1086), rabbit anti-GFP (1:500, ThermoFisher, A11122), chicken anti-GFP (1:1000, Abcam, ab13970), goat anti-ICAM1 (1:500, R&D, AF796), rabbit anti-Cytokeratin 14 (1:200, Proteintech, 10143-1-AP), rabbit anti-P63 (1:100, Abcam, ab63881), mouse anti-Cytokeratin 18 (1:100, Abcam, ab668), goat anti-DCX (1:200, Santa Cruz, sc-8066), rabbit anti-OMP (1:200, Abcam, ab183947) and mouse anti-Tuj1 (1:200, Abcam, #ab78078). Tissue sections were then incubated with secondary antibodies at room temperature for 1 h. The secondary antibodies (1: 300, ThermoFisher) included donkey anti-goat-568, donkey anti-mouse-568, donkey anti-rabbit-568, donkey anti rabbit-647, donkey anti-mouse-488, and donkey anti-goat-633. Tissues were mounted in Vectashield (Vector Laboratories). Fluorescent images were taken under a SP5/Leica confocal microscope with LAS AF Lite software. All primary antibodies were listed in Table S1.

### RNAscope

The procedure was followed by the RNAscope kit (Bio-techne, #323100-USM) manual. Briefly, OE sections were rinsed in 50%, 70% ,100% ethanol and PBS to remove OCT and pretreated with RNAscope hydrogen peroxide, target repair reagent and protease Ⅲ. Then, 4-6 drops of Lgr5 probe (Bio-techne, #312171) were added and the sections were incubated at 40 ℃ for 2 h in HybEZ^TM^ hybridization oven. After rinsed twice with fresh washing buffer, 4-6 drops of multichannel second-generation fluorescent AMP1 were added, and the sections were incubated at 40 ℃ for 30 min in HybEZ^TM^ hybridization oven. Similarly, the sections were hybridized with AMP2 and AMP3. This was followed by signal labeling of C1 channel probe, with 4-6 drops of multichannel second-generation fluorescent HRP-C1 were added, and the OE sections were incubated at 40 ℃ for 15 min in HybEZ^TM^ hybridization oven. Sections were incubated with 150-200 µL diluted TSA Plus fluorescent dye at 40 ℃ for 30 min. Finally, tissues were covered with 4-6 drops of multichannel second-generation fluorescent HRP blocker at 40 ℃ for 15 min. The immunohistochemistry was carried out as above mentioned if required. Tissues were mounted in Vectashield (Vector Laboratories). Fluorescent images were taken under a SP5/Leica confocal microscope with LAS AF Lite software.

### Lentiviral injection

The shLgr5-mCherry lentivirus targeting the mouse Lgr5 was prepared by GENECHEM (Shanghai, China). Lentivirus expressing scramble shRNA (Lenti-shCtrl) was used as negative control. The viral infection was followed by our established procedure 16. Briefly, 5 μL saline solution containing 1x10^6^ TU lentivirus was injected into each side of OE in wide type C57BL/6J mice at three-month-old age, using 1 μL microsyringe (Fine Science Tools, CA, USA) on stereotaxic instrument (RWD, Shenzhen, China). The efficiency of Lenti-shLgr5 was validated in our previous study 41, by qPCR and RNAscope. Methimazole was administrated on Day 10 after viral injection.

### Quantitative real-time PCR

Total RNA was extracted from the Lenti-shCtrl- and Lenti-shLgr5-infected OE tissues by using the E.Z.N.A. Total RNA Kit I (catalog #R6834-02, Omega) according to the manufacturer manual. The extracted RNA was immediately dissolved in RNase-free water, and the purity and concentration were determined using a BioPhotometer (Metash). First-strand cDNA was synthesized using a PrimeScript RT Master Mix (catalog #RR036A, Takara). Primers used in this study were synthesized by Ruidibio. Quantitative real-time PCR was performed on an Analytik Jena Real-Time PCR System. The reaction mixtures included a cDNA template, 0.2 mM primers, SYBR qPCR SuperMix (catalog #E096-01B, Novoprotein), and double distilled H_2_O. Reaction conditions included an initial denaturation at 95 ℃ for 1 min, followed by 40 cycles of 95 ℃ for 20 s, 60 ℃ for 20 s, and 72 ℃ for 30 s. The relative expression levels were calculated using the 2^-△△Ct^ method. Primer sequences were as follows: Lgr5: TAAAGACGACGGCAACAGTG and GATTCGGATCAGCCAGCTAC. GAPDH: TCAATGAAGGGGTCGTTGAT and CGTCCCGTAGACAAAATGGT.

### Quantitative analysis

Cell counts were corrected using Abercrombie's formula: Corrected number = Count × [section thickness / (section thickness + mean nuclei size)]. Counting was carried out by someone who was blind to the experimental design to eliminate bias. Data were presented as mean ± SEM from three independent experiments. Quantitative difference was determined by student's t tests, one-way ANOVA and two-way ANOVA using GraphPad Prism 8.02 software.

## Results

### Lgr5 marks HBCs in the adult OE

Previous work demonstrated that Lgr5 marked mitotically active GBCs in the OE 37. Here, we determined whether Lgr5 marked HBCs in the OE using Lgr5-EGFP-Cre^ERT2^ mice. In animals at postnatal day 1, Lgr5^+^ cells in the OE expressed basal cell marker Sox2, showing the morphology of GBC (Figure S1A, marked by arrowheads). In the OE of mice at three-month-old age, Lgr5-GFP^+^/Sox2^+^ basal cell also represented typical morphology of GBC (arrowhead in Figure S1B). Quantitative analysis showed that the Lgr5^+^ basal cells were present at embryonic day 16 (E16), and the number per 0.5mm OE section continued to rise until postnatal day 1. Then, the number of Lgr5^+^ basal cell decreased and maintained at ~4 cells per 0.5 mm OE section in one- and three-month-old animals, as shown in Figure S1C. These data suggested that Lgr5 expression level in the OE was at the peak around neonatal stage. To further characterize the Lgr5^+^ cells in the OE, we immunostained Lgr5-GFP^+^ cells in the OE of mice at P1 and 3-month-old age against GBC and HBC molecular markers. At P1 and 3-month-old age animals, Lgr5-GFP^+^ cells in the OE expressed NeuroD1, suggesting they were immediate neuronally specified precursor cells (Figure S1D-E, marked by arrowheads). Besides, a small portion of Lgr5-GFP^+^ cells were positively immunostained with Krt14 in the adult OE, demonstrating these Lgr5^+^ cells were HBCs (Figure S1G-G', arrowheads-marked). However, these Lgr5-GFP^+^/Krt14^+^ cells did not possess typical flat morphology. By contrast, we did not observe Lgr5-GFP^+^/Krt14^+^ cell in the OE of P1 age mice, showing all Lgr5^+^ cells were mitotically active at this stage (Figure S1F-F'). We then used RNAscope in situ analysis to further validate Lgr5 expression in the OE of newborn and adult mice, and found apparent Lgr5-mRNA expression across the OE at P1 while the expression was less significant in the adult OE (Figure S2A-B). Lgr5-mRNA expression was beyond the basal cell layer in the neonatal OE, mainly localized in the apical cell layer (Figure S2B), and visible Lgr5-mRNA^+^ signals were also present in the OE apical layer of mice at 3-month-old age (Fig. S2A), but much less apparent compared to that in the neonatal OE. Lgr5-mRNA was expressed in IL33^+^ supporting cells in the OE of either newborn or adult, while Lgr5-mRNA expression was more abundant in the basal cell layer of neonatal OE (Figure S2C-C', E-E'). Consistent with the presence of Lgr5-GFP^+^ HBCs in the adult OE, Lgr5-mRNA was expressed in ICAM1^+^ HBCs of mice at 3-month-old age (Figure S2D-D'), but seldom in the OE of newborns (Figure S2F-F'). By contrast, OMP^+^ sensory neurons in the OE of neither newborns nor adults expressed Lgr5-mRNA (Figure S2D, F). Thus, Lgr5 marks HBCs in the OE at adult but not neonatal stage.

### Lgr5 expression in apical cell layer of dorsal OE post injury

**Figure 1:**
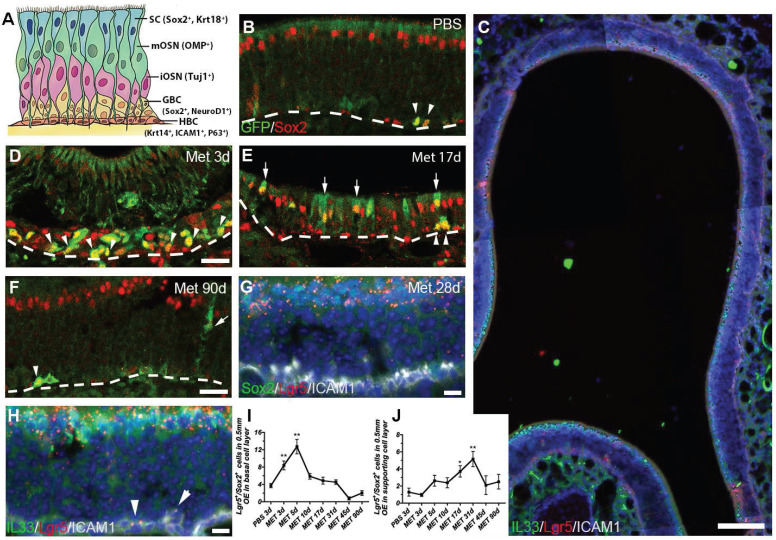
Lgr5 marks both basal and supporting cells in the injured OE. (A) Schematic of the OE containing multiple cell types with biomarkers in parentheses. (B, D-F) Immunostaining against GFP and Sox2 in the OE of saline-injected Lgr5-EGFP-Cre^ERT2^ mice (B) and animals at Day 3, 17 and 90 post injury (D-F). (C, G, H) RNAscope in situ analysis on Lgr5-mRNA and immunostaining against Sox2, ICAM1, IL33 in the OE at Day 28 post injury. (I, J) Quantitative analysis on Lgr5-GFP^+^/Sox2^+^ cell density (the cell number per 0.5 mm OE in each section) in basal and non-basal cell layer of injured OE. Mathimazole was injected into mice at 2-month-old age. Lgr5-GFP^+^/Sox2^+^ cells were noted by arrowheads in basal cell layer and by arrows in supporting cell layer in (B, D-F). Lgr5-mRNA^+^/ICAM1^+^ cells were labeled by arrowheads in (H). Dashed line represented the basal lamina. Quantitative significance was determined by one-way ANOVA with Dunnett's multiple comparisons test, compared to the saline control. * p < 0.05, ** p < 0.01. Scale bars, 50 μm in (C), 25 μm in (D, F), 10 μm in (G, H).

The OE is a pseudostratified epithelial structure composed of several cellular subtypes (Figure 1A). To comprehensively explore the role of Lgr5+ basal cells in the OE regeneration, the whole epithelium except for the remaining HBCs of Lgr5-EGFP-IRES-Cre^ERT2^ mice was wiped out by methimazole (Met) treatment and then we determined Lgr5^+^ cell recruitment in different OE zones. At Day 3 post injury, the Lgr5-GFP^+^ cells were present throughout the OE and most Lgr5-GFP^+^ cells were Sox2^+^ (Figures S3A and 1D). Interestingly, the Lgr5-GFP^+^ cells were abundantly localized in the dorsal zone at Day 10, 17 and 31 post injury (Figure S3B-D, S3B'-D', asterisk-labeled). In the dorsal OE, apical Lgr5-GFP^+^/Sox2^+^ cells were present at Day 17 post injury (Figure 1E, arrow-labeled). RNAscope in situ analysis also confirmed the abundance of Lgr5-mRNA^+^ cells in the apical layer of dorsal OE at Day 28 post injury (Figure S3G-G'). The presence of Lgr5-mRNA^+^/Sox2^+^ and Lgr5-mRNA^+^/IL33^+^ supporting cells in the dorsal OE at Day 28 was detected by RNAscope, indicating Lgr5 marked apical supporting cells during regeneration (Figure 1C, G, H). Furthermore, a few Lgr5-mRNA^+^/ICAM1^+^ cells were observed at this stage, indicating the presence of Lgr5-mRNA^+^ HBCs in the regenerated OE (arrowheads in Figure 1H). Quantitative data showed the number of Lgr5-GFP^+^/Sox2^+^ basal cells per 0.5mm linear length OE in each section was increased to ~8 and ~12 at Day 3 and Day 5 post injury (p < 0.01), and decreased gradually to threshold level from Day 10 to Day 90, similar as the number in saline control (Figure 1B, D-F, I). By contrast, Lgr5-GFP^+^/Sox2^+^ cells outside the basal layer were obviously present in the OE (mostly in the apical layer of dorsal zone) at Day 17 and Day 31 post injury, and then decreased to threshold level at Day 90 (Figure 1E, F, J, p < 0.05 at Day 17 and p < 0.01 at Day 31, labeled by arrows in Figure 1E). These observations suggest a dynamic recruitment of Lgr5^+^ cells during the OE regeneration, while apical Lgr5^+^ cells in the dorsal OE may contribute to the regeneration.

### Lgr5 marks HBCs and sensory neurons during OE regeneration

**Figure 2:**
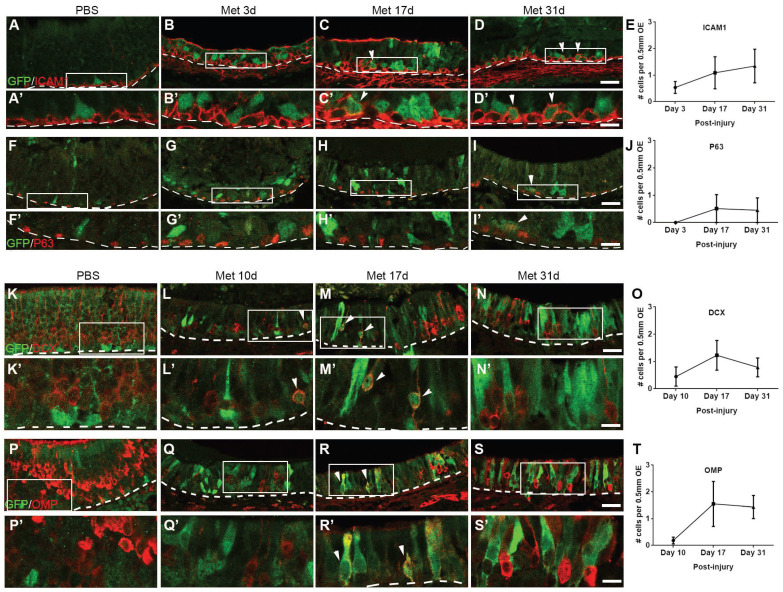
Lgr5 marks HBCs and is transiently expressed in sensory neurons in the injured OE. Confocal images of immunostaining against GFP and ICAM1(A-D) or GFP and P63 (F-I) in the OE of saline- and methimazole-injected Lgr5-EGFP-Cre^ERT2^ mice at Day 3, 17 and 31 post injury. Lgr5-GFP^+^/ICAM1^+^ and Lgr5-GFP^+^/P63^+^ cells were noted by arrowheads. Confocal images of GFP^+^ and DCX^+^ (K-N) or GFP^+^ and OMP^+^ cells (P-S) in the OE of saline-injected controls and mice at Day 10, 17 and 31 post injury. Lgr5^+^/DCX^+^ and Lgr5^+^/OMP^+^ cells were labeled by arrowheads. (E, J, O, T) Quantitative analysis on the number of Lgr5-GFP^+^/ICAM1^+^, Lgr5-GFP^+^/P63^+^, Lgr5-GFP^+^/DCX^+^ or Lgr5-GFP^+^/OMP^+^ cells in the injured OE. Mathimazole was injected into mice at 2-month-old age. Boxed areas in (A-D, F-I, K-N, P-S) were highlighted as (A'-D', F'-I', K'-N', P'-S'). Dashed line represented the basal lamina. Scale bars in (D, I, N, S) were 25 μm, and in (D', I', N', S') were 10 μm.

According to the previous report, Lgr5^+^ cells mobilized in the injured OE were GBCs 37. Besides, the presence of Lgr5-mRNA^+^/ICAM1^+^ cells post OE injury was validated by RNAscope (Figure 1H, arrowheads-labeled). To further characterize it, OE sections of Lgr5-EGFP-Cre^ERT2^ mice were then immunostained with antibodies against HBC marker P63 and ICAM1. Almost all Lgr5-GFP^+^ basal cells did not express ICAM1 or P63 at Day 3 post injury (Figure 2B-B', G-G'). By contrast, we observed the presence of Lgr5-GFP^+^/ICAM1^+^ cells at Day 17 and 31 post injury (Figure 2C-C', D-D', marked by arrowheads, ~2 cells per 1 mm OE in each section) and Lgr5-GFP^+^/P63^+^ cells at Day 31 (Figure 2I-I', marked by arrowhead, ~1 cell per 1 mm OE section). Considering that most Lgr5^+^ cells were GBCs in the injured OE and a very small portion of them were HBCs, we hypothesized that Lgr5^+^ cells were recruited to active state by injury, while a few of them returned to dormancy in the recovered OE. More strikingly, some Lgr5-GFP^+^ cells not localized in the basal cell layer could express immature and mature neuronal markers in the injured OE. At Day 10 post injury, ~2 Lgr5-GFP^+^ cells per 1 mm OE section expressed neuronal progenitor and immature neuronal marker DCX, and ~3 Lgr5-GFP^+^/DCX^+^ cells per 1 mm OE section were observed at Day 17 (Figure 2L-L', M-M', O, marked by arrowheads). However, there was no Lgr5-GFP^+^/DCX^+^ cell in the OE of saline-injected controls (Figure 2K-K'). Similarly, ~3 Lgr5-GFP^+^/OMP^+^ cells per 1 mm OE section were present at Day 17 (Fig.2R, R', T, marked by arrowheads). However, there was no Lgr5-GFP^+^/OMP^+^ cell in saline controls or in animals at Day 10 post injury (Figure 2P, Q). Collectively, these data demonstrated that most of recruited Lgr5^+^ cells in the injured OE were proliferative neuronal-specified progenitors, while a small portion of Lgr5^+^ cells were HBCs when the OE was almost fully regenerated. A few Lgr5^+^ cells underwent a transient and intermediate state to express the neuronal markers, potentially suggesting a role of Lgr5 in sequential differentiation from HBC to neuronal progenitor, immature sensory neurons, and mature neurons.

### Lgr5^+^ cells function as unipotent cells in the neonatal OE but multipotent cells in the injured OE

To further elucidate whether Lgr5^+^ cells played the different roles in the OE of neonatal, adult, and injured animals, we lineage-traced Lgr5^+^ cells using Lgr5-Cre^ERT2^/Rosa26-TdTomato mice through tamoxifen induction. Firstly, we induced the generation of Cre in 3-month-old mice by three consecutive doses of tamoxifen (Figure S4A), which were sufficient to Cre induction and generation of TdTomato^+^ cells. Three weeks after induction, we only observed very limited number of TdTomato^+^ cells throughout the OE, demonstrating that Lgr5^+^ cells were not consistently active in the OE homeostasis at adult stage (Figure S4C). This was supported by the finding that limited number of Lgr5-mRNA^+^ cells in the GBC layer (Figure S2C-D), while Lgr5-mRNA^+^ HBCs were present in the adult OE (Figure S2D-D'). Meanwhile, Lgr5-GFP^+^ cells were also sparse throughout the OE (Figure S4C'). Similar result was obtained at Day 90 after tamoxifen induction, suggesting that inefficiency in generating TdTomato^+^ progenies from lineage-traced Lgr5^+^ cells was not due to the insufficient tracing duration. Then, we injected one dose of tamoxifen into P0 mice to investigate whether Lgr5^+^ cells generated progenies in the OE from P1 to 1-month-old age (Figure S4B). TdTomato^+^ cells were scattered across the epithelium (Figure S4D) and TdTomato^+^ cell bundle was also present in the OE at Day 7 after lineage tracing (arrowhead in Figure S4D), although neither TdTomato^+^ cell nor cell bundle was abundant. These TdTomato^+^ cells were not GFP^+^, showing that they were daughter cells generated from Lgr5^+^ cells (Figure S4D'). Compared to Lgr5^+^ cells in adult mice, lineage-traced Lgr5^+^ cells from P1 age generated 1.6 ± 0.4 folds more TdTomato^+^ cells (7 ± 1 cells per 0.5 mm OE in each section) at Day 7 after induction (Figure S4E). At three weeks after tamoxifen injection, TdTomato^+^ cells in the OE of adult mice did not localize in apical supporting cell layer (Figure S4G), while a few Tuj1^+^ immature neurons but not OMP^+^ mature neurons were generated from Lgr5+ cells in the OE (Figure S4J, M). Lgr5^+^ cells lineage-traced at P1 also did not generate supporting cell at Day 7 post tamoxifen injection (Figure S4E, H), while TdTomato^+^/Tuj1^+^ immature neurons and a few TdTomato^+^/OMP^+^ mature neurons were present at Day 7 (Figure S4E, K, N), and 2.0 ± 0.4 TdTomato^+^/DCX^+^ cells were present along 0.5mm OE on each section at Day 7 (Figure S4E). By contrast, TdTomato^+^/OMP^+^ neurons were observed at Day 31 when Lgr5^+^ cells were lineage-traced at P1 (Figure S4I), and TdTomato^+^ signals were also found in OMP^+^ cilia (Figure S4L), showing that Lgr5^+^ cells traced at newborn stage were able to generate mature OSNs at Day 31 post lineage tracing. These data indicated that Lgr5^+^ cells in the uninjured neonatal OE functioned as GBCs but not multipotent HBCs since only sensory neurons were derived from Lgr5^+^ cells.

To show the difference in progenies generated from Lgr5^+^ cells between OE homeostasis and regeneration, we lineage-traced Lgr5^+^ cells in the injured OE. As shown in Figure S4O, single dose of methimazole (or saline as control) was administrated, followed by three consecutive doses of tamoxifen. Compared to the uninjured control (Figure S4R, R”), injury led to more abundant generation of TdTomato^+^ cells throughout the OE after tamoxifen administration, suggesting active participation of Lgr5^+^ cells in the OE regeneration (Figure S4Q, Q”). At Day 7 post tamoxifen induction (equaling Day 11 post OE injury), the number of TdTomato^+^ cells in 0.5mm OE per section with three doses of tamoxifen (16 ± 1 TdTomato^+^ cells) was not significantly different from the number with single dose of tamoxifen (14 ± 1 TdTomato^+^ cells) (Figure S4F, P, Q), suggesting one dose of tamoxifen was also sufficient to Cre induction. 83±4% of TdTomato^+^ cells in injured OE at Day 7 post induction were not GFP^+^, showing the majority of TdTomato^+^ cells were generated from Lgr5^+^ cells (Figure S4P', Q'). TdTomato^+^ cells were located at different layers in the lesioned OE, indicating injury led to the multipotency of Lgr5^+^ cells (Figure S4P”, Q”). However, TdTomato^+^ cells were sparse in the uninjured OE (Figure S4R, R”). Quantitatively, the number of TdTomato^+^ cells per 0.5 mm length OE in each section at Day 7 post tamoxifen induction was significantly increased by 4.2 ± 0.5 and 2.9 ± 0.3 folds in the lesioned OE than in the OE of saline-injected controls, when one dose or three doses of tamoxifen were administrated (Figure S4F, p < 0.001). Furthermore, TdTomato^+^ cells expressed apical supporting cell marker Sox2 (2 ± 1 cells per 0.5 mm OE in each section) and mature sensory neuron marker OMP (12 ± 4 cells per 0.5 mm OE in each section) in the injured OE at Day 21 after tamoxifen induction (Figure S4F, S, T). However, we did not observe any co-localization of TdTomato with HBC marker ICAM1 (Figure S4U), suggesting that Lgr5^+^ progenitors did not generate HBCs at Day 21. Meanwhile, most of TdTomato^+^/Sox2^+^ or TdTomato^+^/OMP^+^ cells did not express GFP, indicating that they were derived from Lgr5^+^ cells during OE regeneration (Figure S4S', T'). Collectively, these data demonstrated that Lgr5^+^ cells participated in OE homeostasis from neonatal to youth, and in regeneration of the injured adult OE, functioning as unipotent GBCs and multipotent cells, respectively, but did not undergo persistent differentiation in the uninjured OE of adults.

### Lgr5^+^ cells generate HBCs when the OE recovers from injury

**Figure 3:**
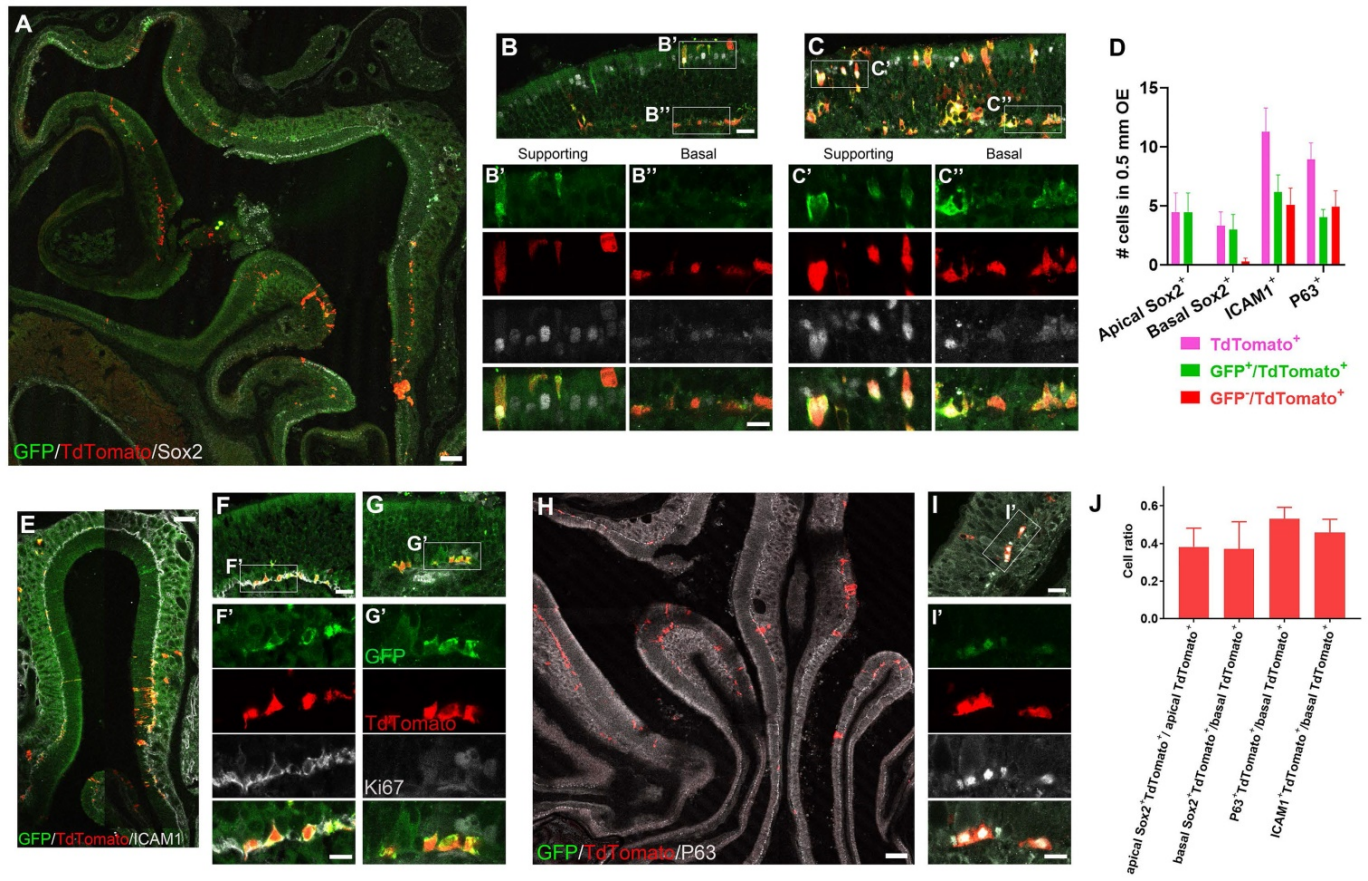
Lgr5^+^ cells produce HBCs in the regenerated OE. (A-C) Immunostaining against GFP and Sox2 in the OE of methimazole-injected Lgr5-EGFP-Cre^ERT2^/Rosa26-fl-STOP-fl-TdTomato (LT) mice at Day 90 post three doses of tamoxifen induction. (D) Quantitative analysis on the Lgr5-GFP^+^ and Lgr5-GFP^-^ /TdTomato^+^ cells, including Sox2^+^ supporting and basal cells, ICAM1^+^ or P63^+^ HBCs. (E-I) Confocal images of ICAM1^+^/TdTomato^+^ (E, F), Ki67^+^ (G) or P63^+^/TdTomato^+^ cells (H, I) in the OE of LT mice at Day 90 after tamoxifen induction. (J) Quantitative analysis on the ratio of Sox2^+^, ICAM1^+^ or P63^+^ /TdTomato^+^ cells. Mathimazole was injected into mice at 3-month-old age. Boxed regions in (B, C, F, G, I) were highlighted as (B', B'', C', C'', F', G', I'). Scale bars in (A, E, H) were 100 μm, in (B, F, I) were 25 μm, and in (B'', F', I') were 10 μm.

Next, we determined whether Lgr5^+^ cells derived or became HBCs when OE was regenerated. Three months after tamoxifen induction, TdTomato^+^ cells expressed Sox2 in both apical supporting and basal cells (Fig. 3A). In both TdTomato^+^ cell-sparse (Figure 3B) and -abundant (Figure 3C) zones, a few apical Sox2^+^TdTomato^+^ cells were GFP^+^ (Figure 3B', C', D), while 12.5 ± 4.4% of Sox2^+^/TdTomato^+^ basal cells did not express GFP (Figure 3B, B'', C, C'', D). The presence of Sox2^+^/GFP^+^/TdTomato^+^ cells suggested that Lgr5^+^ supporting and basal cells were present in the regenerated OE. Interestingly, 45.9 ± 7.0% of TdTomato^+^ cells in the basal cell layer were co-localized with HBC markers ICAM1 (Figure 3E, F, F', J), and 42.8 ± 9.7% of TdTomato^+^/ICAM1^+^ cells did not express GFP (Figure 3D, F'), showing that these GFP^-^/TdTomato^+^/ICAM1^+^ cells were derived from Lgr5^+^ cells, further suggesting that Lgr5^+^ cells generated HBCs in the recovered OE. Furthermore, Lgr5^+^/TdTomato^+^ cells in the basal cell layer did not express Ki67, indicating these cells were not proliferative in the regenerated OE at three-months post injury (Figure 3G-G'). Consistent with immunostaining data against ICAM1, we also found that 53.0 ± 6.1% of TdTomato^+^ basal cells expressed P63 in the OE at Day 90 post tamoxifen induction (Figure 3H-J), while 56.5 ± 8.4% of TdTomato^+^/P63^+^ cells were GFP^+^ (Figure 3D, H, I, I'), strongly supporting our conclusion that a few Lgr5^+^ cells or progenies from Lgr5^+^ cells were HBCs in the regenerated OE. By contrast, we did not observe any TdTomato^+^/ICAM1^+^ cells at Day 21 post tamoxifen induction (Figure S4U), suggesting that Lgr5^+^ cells were active and did not generate HBCs at this stage. Collectively, these data demonstrated that Lgr5^+^ cells participated in the OE regeneration and generated HBCs after tissue recovery from injury.

### Lgr5^+^ cells are necessary in the regeneration of injured OE

We then asked whether Lgr5^+^ cells were required in OE homeostasis or regeneration. Tamoxifen and diphtheria toxin (DTX) were injected into Lgr5-EGFP-Cre^ERT2^/Rosa26-iDTR mice to selectively deplete Lgr5^+^ cells. We firstly explored whether Lgr5^+^ cells were required in the uninjured OE of 3-month-old mice. As indicated in the scheme (Figure S5A), mice were given five doses of tamoxifen and five doses of DTX in ten consecutive days. Animals were sacrificed at Day 17, 31 and 60 post the first dose of tamoxifen. We did not trace longer time since five doses of DTX were not sufficient to deplete Lgr5^+^ cells lasting for more than two months while more than five doses of DTX led to high mortality. We did not find significant alteration in cellular composition throughout the epithelium during two months after DTX administration, and the data collected at Day 31 was shown as Figure S5, demonstrating that no obvious alteration was found in the density of OMP^+^ mature sensory neurons (Figure S5B, C) or Sox2^+^ supporting and basal cells (Figure S5D, E). Besides, the OE thickness was not changed with the depletion of Lgr5^+^ cells at Day 60 (Figure S5F). These data suggested that Lgr5^+^ cells were not necessary in the homeostasis of adult OE, at least during 60 days after Lgr5^+^ cell depletion.

**Figure 4:**
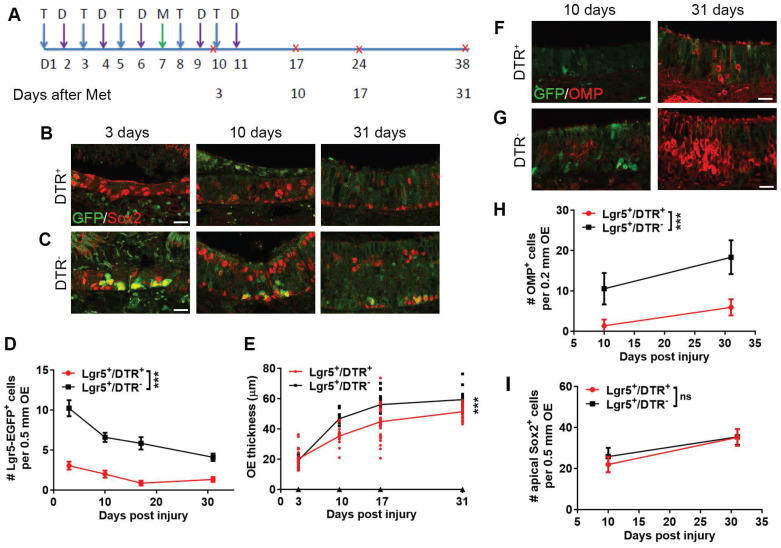
Lgr5^+^ cells are required in the OE regeneration. (A) Scheme showing the tamoxifen (abbreviated as T), diphtheria toxin (abbreviated as D) and methimazole (abbreviated as M) injection into Lgr5-EGFP-Cre^ERT2^/Rosa-fl-STOP-fl-DTR (Lgr5^+^/DTR^+^) mice. (B, C) Confocal image of Lgr5-GFP^+^ and Sox2^+^ cells in the OE of Lgr5-Cre^ERT2^/Rosa-DTR^+^ and Lgr5-Cre^ERT2^/Rosa-DTR^-^ mice at Day 3, 10, 31 post injury. (D) Quantitative analysis of Lgr5-GFP^+^ cells in the OE of Lgr5-Cre^ERT2^/Rosa-DTR^+^ and Lgr5-Cre^ERT2^/Rosa-DTR^-^ mice at Day 3, 10, 31 post injury. (E) Quantitative analysis of OE thickness. (F, G) Confocal images of Lgr5-GFP^+^ and OMP^+^ cells in the OE of Lgr5-Cre^ERT2^/Rosa-DTR^+^ and Lgr5-Cre^ERT2^/Rosa-DTR^-^ mice at Day 10, 31 post injury. (H, I) Quantitative analysis of OMP^+^ and apical Sox2^+^ cells at Day 10, 31 post injury. Mathimazole was injected into mice at 3-month-old age. Statistical significance was measured by two-way ANOVA with Sidak's multiple comparisons test. ns, not significant, *** p < 0.001. Scale bars: 10 µm.

Then, we elucidated if Lgr5^+^ cell participation was important for OE regeneration. Lgr5^+^ cells were eradicated in the lesioned OE and mice were sacrificed at Day 3, 10, 17 and 31 post injury (Figure 4A). The number of Lgr5^+^ cells (per 0.5 mm OE in each section) from Day 3 to 31 post injury was dramatically decreased in Lgr5-EGFP-Cre^ERT2^/Rosa-DTR^+^ mice compared to Lgr5-EGFP-Cre^ERT2^/Rosa-DTR^-^ mice with tamoxifen and DTX administration (p < 0.001, Figure 4B-D). Furthermore, the OE thickness in Lgr5-EGFP-Cre^ERT2^/Rosa-DTR^+^ mice was decreased by 24.7 ± 4.3%, 25.4 ± 1.7% and 15.6 ± 3.2% at Day 10, 17 and 31 post injury (p < 0.001), but not significantly altered at Day 3 compared to Lgr5-EGFP-Cre^ERT2^/Rosa-DTR^-^ mice (Figure 4E). Then, we determined whether Lgr5^+^ cells were necessary to reconstruct different cell layers in the injured OE. At Day 10 and 31 post injury, apparent supporting cells and mature OSNs were present in Lgr5-EGFP-Cre^ERT2^/Rosa-DTR^-^ mice (Figure 4C, G). By contrast, we observed supporting cells in Lgr5-EGFP-Cre^ERT2^/Rosa-DTR^+^ mice while the mature OSNs were sparse (Figure 4B, F). Quantitatively, the number of OMP^+^ mature OSNs along 0.2 mm OE in each section was decreased by 87.2 ± 14.6% and 67.7 ± 10.9% at Day 10 and 31 (p < 0.001, Figure 4H), while the number of supporting cells was not significantly altered with depletion of Lgr5^+^ cells in the OE (p > 0.1, Figure 4I). Thus, Lgr5^+^ cells were important to the neuronal regeneration in the injured OE.

**Figure 5:**
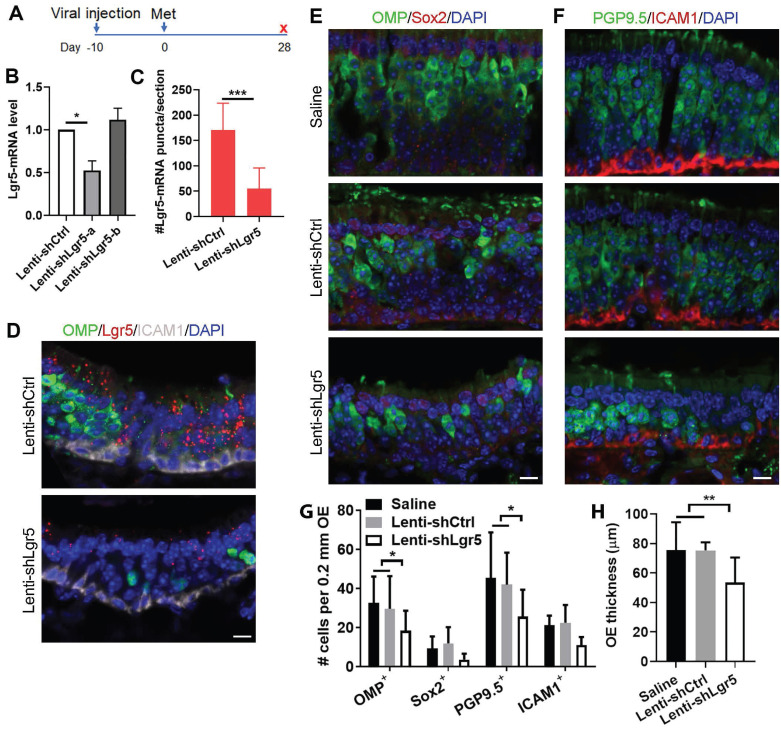
Lgr5 downregulation impairs sensory neuron regeneration in injured OE. (A) Scheme showing the lentiviral and methimazole injection in wide type C57BL/6J mice at three-month-old age. (B) Quantitative PCR on Lgr5-mRNA level in the OE infected with Lenti-shCtrl, Lenti-shLgr5-a or Lenti-shLgr5-b. (C) Quantitative analysis on Lgr5-mRNA^+^ signals in Lenti-shCtrl or Lenti-shLgr5-infected OE by RNAscope technique. (D) Confocal images of Lgr5-mRNA^+^ cells in the OE infected with Lenti-shCtrl and Lenti-shLgr5. (E, F) Confocal images of OMP^+^, Sox2^+^, PGP9.5^+^ and ICAM1^+^ cells in the OE of mice receiving saline, Lenti-shCtrl or Lenti-shLgr5. (G) Quantitative analysis on the numbers of OMP^+^, Sox2^+^, PGP9.5^+^ and ICAM1^+^ cells in mice injected with saline, Lenti-shCtrl or Lenti-shLgr5. (H) Quantitative analysis on OE thickness. The significance was determined by unpaired t test in (B, C, H) and by two-way ANOVA with Dunnett's multiple comparisons test in (G). Scale bars: 10 µm.

To further demonstrate the role of Lgr5 in the OE regeneration, we then injected lentivirus expressing Lgr5-shRNA into the OE ten days before methimazole administration (Figure 5A). At Day 28 post injury, the Lgr5-mRNA level in the OE infected with Lenti-shLgr5-a was reduced by 48 ± 8% compared to infection with Lenti-shCtrl, while Lenti-shLgr5-b infection did not affect the Lgr5 expression level in the OE (Figure 5B). RNAscope in situ RNA analysis confirmed that positive signals against Lgr5-mRNA was weaker in Lenti-shLgr5-infected OE compared to Lenti-shCtrl group at Day 28 post injury (Figures 5D and S6). The number of Lgr5-mRNA^+^ puncta on each section of Lenti-shLgr5 group was significantly reduced by 68 ± 8% (Figure 5C, p < 0.001). Thus, we used Lenti-shLgr5-a in the following experiments. In Lenti-shLgr5-infected OE, we found significant decreases in the number of OMP^+^ and PGP9.5^+^ sensory neurons per 0.2 mm OE in each section by 38 ± 11% and 39 ± 14%, compared to animals receiving Lenti-shCtrl (Figure 5E-G, p < 0.05), while Lgr5 downregulation in the OE did not significantly reduce the number of apical Sox2^+^ cells or ICAM1^+^ basal cells (Figure 5E-G, p > 0.05). Lgr5 downregulation led to apparent decrease in OE thickness by 35 ± 7% in mice injected with Lenti-shLgr5 compared to Lenti-shCtrl-injected animals at Day 28 (Figure 5H, p < 0.01). These data indicated that regeneration of OSNs post OE injury was vulnerable to Lgr5 downregulation, suggesting that Lgr5 expression was critical to the neuronal regeneration.

### Olfactory bulbectomy induces the differentiation of Lgr5^+^ cells in the OE

**Figure 6:**
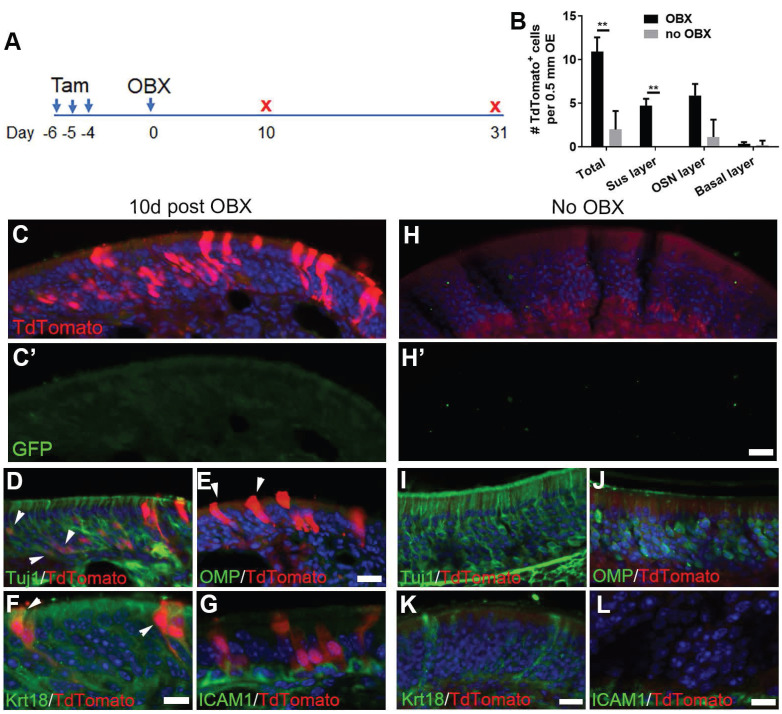
OBX induces multipotency of Lgr5^+^ cells in the OE. (A) Scheme showing the OBX and tamoxifen induction in Lgr5-Cre^ERT2^/Rosa26-TdTomato mice at 3-month-old age. (B) Quantitative analysis on TdTomato^+^ cells in different OE cell layers at either the OBX or no OBX side. (C-G) Confocal images of TdTomato^+^ cells in the OE at Day 10 post OBX. Arrowheads labeled Tuj1+/ TdTomato^+^ and Krt18+/TdTomato^+^ cells in (D) and (F), and TdTomato^+^ supporting cells in (E). (H-L) Confocal images of TdTomato^+^ and Tuj1^+^, OMP^+^, Krt18^+^, ICAM1^+^ cells in the uninjured side at Day 10. (C', H') Images of anti-GFP staining. The significance was determined by unpaired t test in (B), ** p < 0.01. Scale bars, 20 μm.

Since OBX unlocked a multipotent state of neuronal progenitors in the OE 14, we asked whether OBX could recruit the Lgr5^+^ progenitors. The Lgr5-EGFP-Cre^ERT2^/Rosa26-TdTomato mice were subjected to OBX after three doses of tamoxifen induction (Figure 6A). Apparent TdTomato^+^ cells were present at Day 10 post OBX and the number of TdTomato^+^ cells per 0.5 mm OE in each section was increased by 4.4 ± 0.8 folds compared to the uninjured side (no OBX) (Figure 6B, C, H, p < 0.001). These TdTomato^+^ cells in the OE at the OBX side did not express GFP, suggesting these cells were generated from Lgr5^+^ progenitors (Figure 6C'). At Day 10 post OBX, Lgr5^+^ cells generated Tuj1^+^ neurons (Figure 6D, labeled by arrowheads). However, there was no TdToamto^+^/ICAM1^+^ cells (Figure 6G), suggesting that Lgr5^+^ cells did not generate HBCs at Day 10 post OBX. Meanwhile, we did not find any TdTomato^+^/OMP^+^ cells (Figure 6E), showing there was no mature OSNs at the OBX side. TdTomato^+^ cells were localized in the supporting cell layer (arrowheads in Figure 6E and Krt18^+^/TdTomato^+^ cells in Figure 6F), demonstrating that supporting cells were also derived from Lgr5^+^ progenitors when mature neurons were deracinated through OBX. By contrast, in non-OBX side there was abundant Tuj1^+^, OMP^+^, Krt18^+^ or ICAM1^+^ cells but without TdTomato expression (Figure 6I-L). Quantitatively, the number of TdTomato^+^ cells in supporting and neuronal cell layers per 0.5 mm OE in each section was increased from 0 and 1 ± 1 at the non-OBX side to 5 ± 1 and 6 ± 1 at the OBX side, respectively (Figure 6B). Thus, we concluded that OBX could induce Lgr5^+^ progenitors differentiating into immature sensory neurons and supporting cells, demonstrating the multipotent state of Lgr5^+^ cells in the OE under OBX condition.

## Discussion

In this study, we investigate the overall role of Lgr5^+^ cells in the homeostasis and regeneration of the OE. This work shows that Lgr5^+^ cells function as unipotent GBCs in the homeostasis of neonatal OE, while they dynamically participate and are necessary in the OE regeneration serving as multipotent cells. These findings imply potential clinical application of the Lgr5^+^ cell-based therapy against anosmia.

GBCs are proliferative to replenish OSNs in the OE, while the HBCs are recruited and generate various cellular subtypes post injury 11. The lineage tracing analysis in the current study demonstrated that Lgr5^+^ cells were recruited and functioned as multipotent progenitors to generate neuronal and supporting cells during the recovery of lesioned OE, and then served as or generated HBCs in the regenerated OE (Figures 3 and S4). This implies a dynamic role of Lgr5^+^ cells in OE regeneration. These findings strengthen the concept that GBCs are regenerated from HBCs when the OE is severely damaged 9 and support that some GBCs are able to generate HBCs with recovery of the OE. Another interesting finding in this work was that Lgr5 could mark several types of neuronal cells including OMP^+^ as well as DCX^+^ cells at the specific periods during the OE regeneration although the number was limited (Figure 2). It has been reported that the GBCs in the OE have molecular heterogeneity 5. During the progression from progenitor cells to sensory neurons, some molecular identities are altered, including ΔNp63, Sox2, Pax6, Ascl1, Neurog1, NeuroD1, Notch1 5, 10, 42. These may explain our observation that Lgr5^+^/OMP^+^ and Lgr5^+^/DCX^+^ cells were present in the injured OE (Figure 2), potential indicating these cells are transiently present and the expression of Lgr5 may diminish when these neurons become physiologically and functionally mature. Therefore, based on the previous finding demonstrating that Lgr5^+^ cells are GBCs in the OE 37, we systematically disclose their dynamic roles in the regeneration of injured OE.

Since we demonstrated that some Lgr5^+^ cells returned to the quiescence or generated HBCs with the OE recovery, it is plausible that some Lgr5^+^ cells are inactive HBCs in the uninjured OE. In the OE of adult mice at 3-month-old age, a few Lgr5^+^ cells expressed HBC marker Krt14 (Figure S1). However, this was not the case in the P1 mice since we did not observe the expression of HBC markers in Lgr5^+^ cells. The Lgr5^+^/Krt14^+^ cells did not possess the typical morphology of HBCs. One explanation of this scenario is that a small portion of HBCs in the normal OE at adult age are label-retaining and mitotic quiescence. Previous work reported a unique group of cells that were activated in response to epithelial injury and re-establish after the initial phase of recovery is completed 8. This feature is consistent with what we reported here on Lgr5^+^ cells in the OE. Furthermore, lineage tracing in the lesioned OE showed that Lgr5^+^ cells could function as or give rise to HBCs when the OE was regenerated (Figure 3), further suggesting that some Lgr5^+^ cells in the OE may stay at the intermediate state between active GBCs and inactive HBCs. To further validate the idea that whether some Lgr5^+^ cells may function as intermediate quiescent GBCs in the OE, future works will be focused on identifying whether these cells express cyclin-dependent kinase inhibitors and elucidating the origin of Lgr5^+^ cells in the OE.

The OSNs in the nasal cavity are supported by an apical layer of glial-like sustentacular cells 43. In the current study, we observed the presence of Lgr5-GFP^+^/Sox2^+^ cells in the supporting cell layer of dorsal OE during regeneration (Figure 1). These cells were transient in the OE regeneration since most of them disappeared after the OE was completely recovered (Figure 1). Thus, some Lgr5+ cells in supporting cell layer generated during the OE regeneration may undergo a transition from immature to mature state, denoted by the dynamic expression of Lgr5. In the OE from newborn mice, apparent Lgr5-mRNA expression was present in both the apical and basal OE, more significant than in the adult OE (Figure S2). Since the Lgr5^+^ cells in the newborn OE more actively participate in the homeostasis than in the adult OE, it is likely that Lgr5^+^ supporting cells are prone to appear in the developing or regenerating OE. This conclusion will be solidified if specific cellular subtypes that transiently express Lgr5 are identified in the OE. Furthermore, absence of microvillar cells located in the supporting cell layer does not impair the process of neurogenesis 44, implying the unique role of Lgr5^+^ cells in the OE regeneration since depletion of Lgr5^+^ cells or Lgr5 downregulation affected (at least partially) the sensory neuronal regeneration (Figures 4 and 5). More efforts should be focused on the role of zone-specific Lgr5^+^ supporting cells in the process of OE regeneration through coordinating signals from other cellular types in the epithelium.

The pathogenesis of olfactory dysfunction is still not clear and currently there is no efficient therapy against this disease. Aging-induced deficiencies in OE cell proliferation and neuronal differentiation from basal cells lead to olfactory dysfunction 45, 46. Thus, strategies to regulate activation and differentiation of basal cells in the OE will be applicable in the treatment for this disease. In the current study, we explored the function of Lgr5^+^ cells in sensory neuronal regeneration, showing that Lgr5^+^ cells participate in and are vital for OE recovery from injury. Since Lgr5^+^ cells play dynamic roles in OE regeneration, they are ideal candidates for cell-based therapy. Thus, this work provides novel concept for establishing precision medicine against olfactory dysfunction.

## Conclusion

This study presents dynamic roles of Lgr5^+^ cells in the OE homeostasis and regeneration, proposing a new target to induce the sensory neuronal regeneration. Thus, it facilitates us understanding how Lgr5^+^ cells guide the OE regeneration, providing new insight into the therapy against anosmia induced by sensory neuron degeneration.

## Supplementary Material

Supplementary figures and table.
