# Supplementary material for: Lgr5+ cells are required and dynamically participate in olfactory epithelium regeneration: a revisiting shows Lgr5 expression in multiple cell lineages
Source: Theranostics. 2022 Jul 18;12(13):5631–44. doi: 10.7150/thno.60636 (PMC9373817; doi:10.7150/thno.60636)
Supplement: Supplementary file 1 — Supplementary figures and table. [file thnov12p5631s1.pdf]

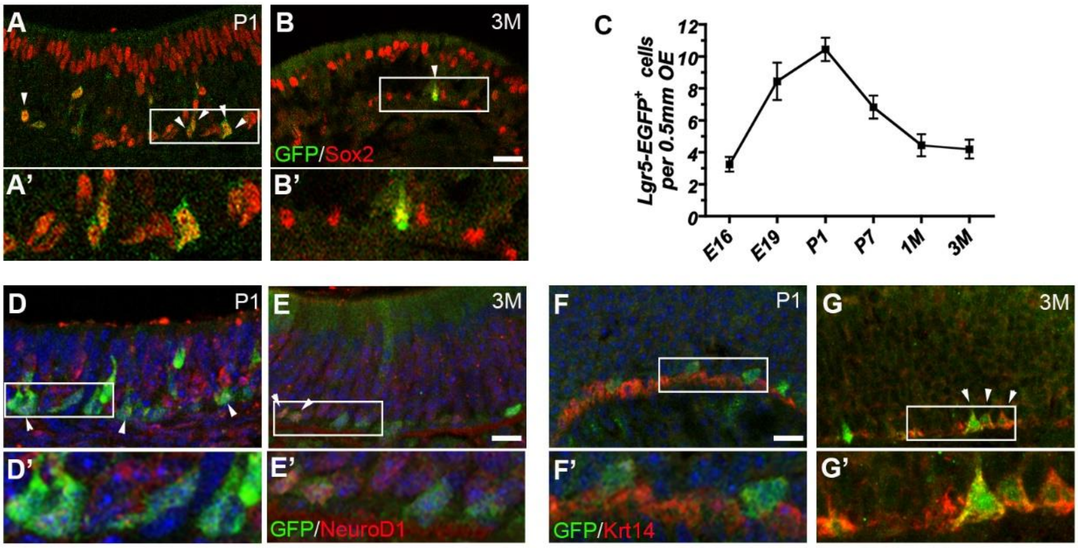

Supplementary Figure 1. Lgr5 marks GBCs and HBCs in the OE. (A, B) Confocal images of Lgr5-GFP$^+$/Sox2$^+$ cells in the OE of P1 and 3-month-old Lgr5-EGFP-Cre^{ERT2} mice. (C) Quantitative analysis on Lgr5-GFP$^+$ cells in the OE. (D, E) Immunostaining against GFP and NeuroD1 in the OE of P1 and 3-month-old mice. (F, G) Confocal images of Lgr5-GFP$^+$ and Krt14$^+$ cells in the OE of mice at P1 and 3-month-old age. Double positively stained cells were indicated by arrowheads. Squared regions in (A, B, D-G) were highlighted as (A', B', D'-G'). Scale bars, 20 µm.

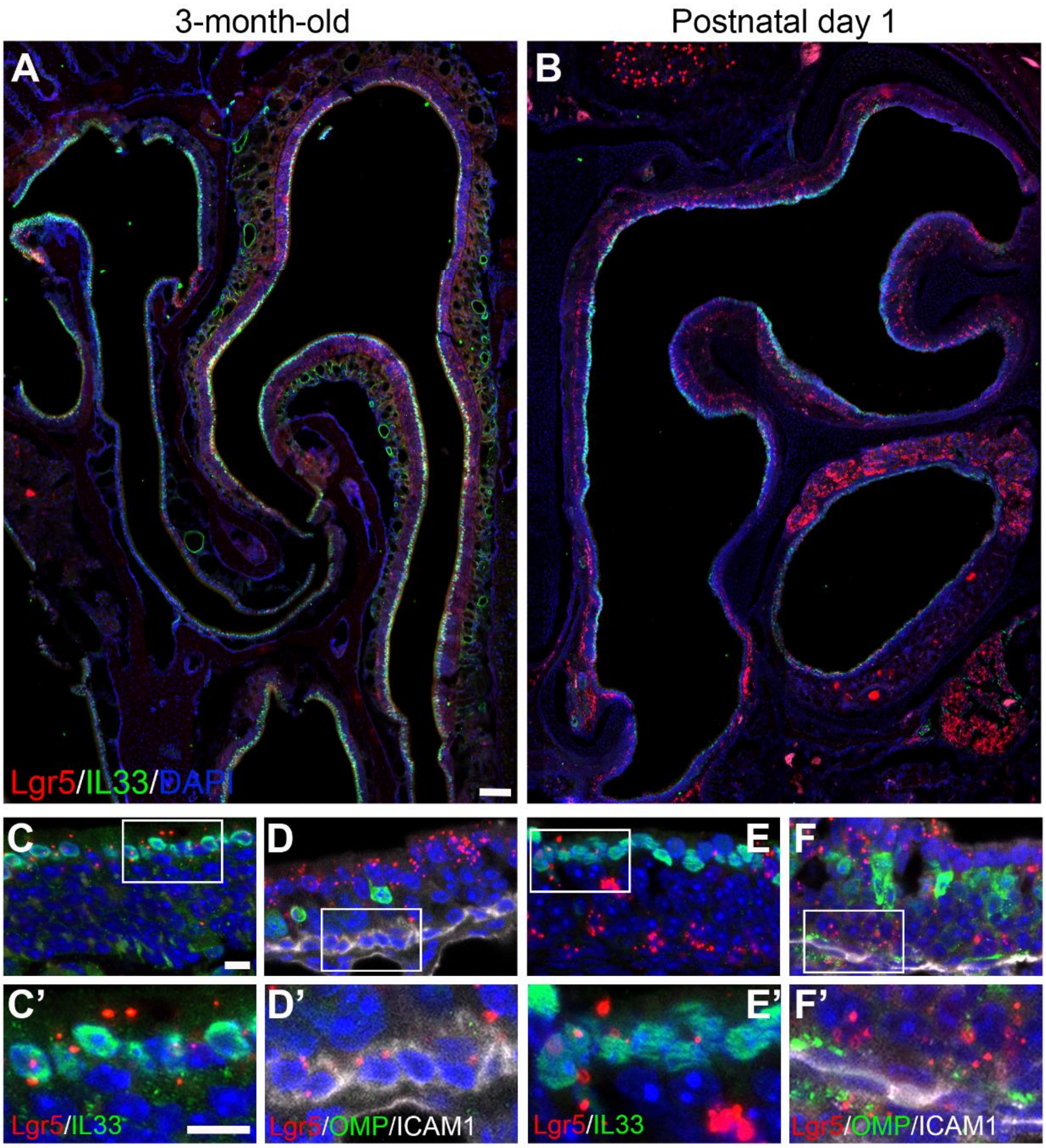

Supplementary Figure 2. Lgr5-mRNA expression in different OE cell lineages. (A, B) Confocal images of Lgr5-mRNA and IL33 expression in the OE of C57BL/6J mice at 3-month-old and P1 age. (C, D) RNAscope analysis on Lgr5 and immunostaining against IL33, OMP, ICAM1 in the adult OE. (E, F) RNAscope and immunostaining in the neonatal OE. (C'-F') were the enlarged rectangular region from (C-F). Scale bars: 100 μm in (A), 20 μm in (C, C').

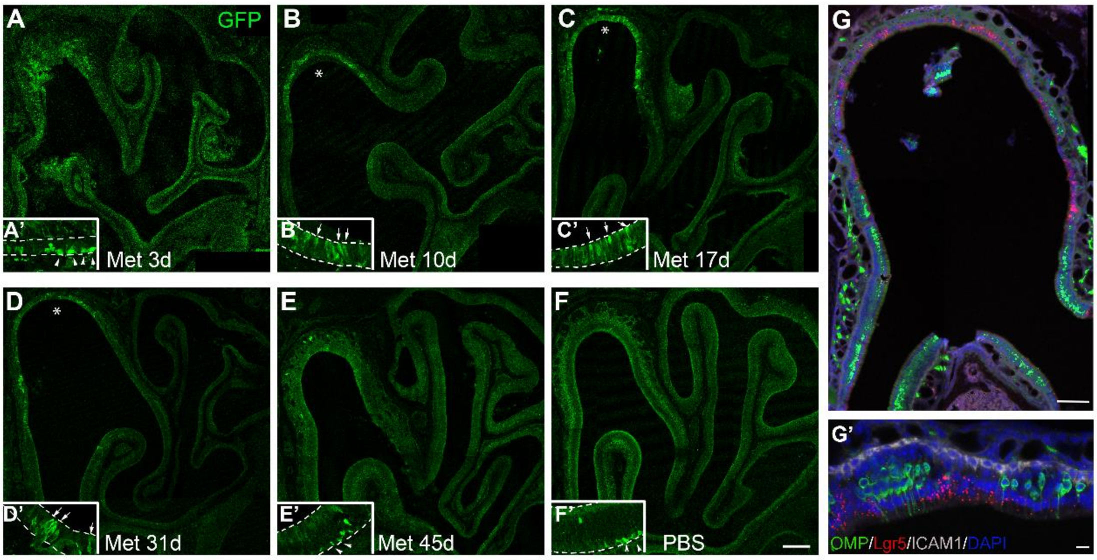

Supplementary Figure 3. Lgr5-GFP$^+$ cells are abundant in apical layer of the injured dorsal OE. (A-F) Confocal images of Lgr5-GFP$^+$ cells in the OE of Lgr5-EGFP-Cre^{ERT2} mice at Day 3, 10, 17, 31 and 45 post injury (A-E) or in the saline control (F). (A'-F') Boxed areas were the partial enlargement. Lgr5-GFP$^+$ cells in basal and non-basal layer were noted by arrowheads and arrows. Asterisks in (B-D) labeled dorsal regions where Lgr5$^+$ cells were abundant at Day 10, 17 and 31 post injury. (G, G') Confocal images of Lgr5-mRNA signals in the OE at Day 28 post injury. Mathimazole was injected into mice at 2-month-old age. The dashed lines represented OE outlines in (A'-F'). Scale bars were 100 μm in (F, G), 10 μm in (G').

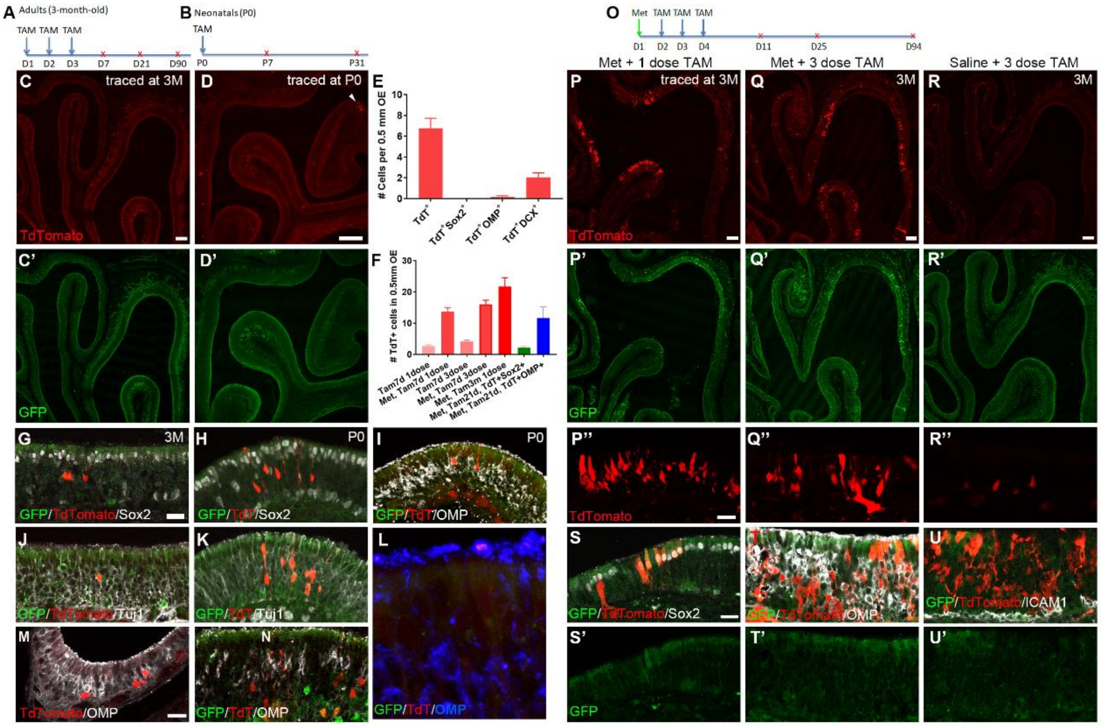

Supplementary Figure 4. Lgr5^{+} cells participate in the neonatal OE homeostasis and are recruited in the injured OE. (A, B) Scheme showing lineage tracing of Lgr5^{+} cells through tamoxifen induction in Lgr5-Cre^{ERT2}/Rosa26-TdTomato (LT) mice at 3-month-old age and postnatal day 0 (P0). Confocal images of TdTomato^{+} (C, D) and GFP^{+} cells (C', D') in the OE of adult (3-month-old) and neonatal mice (P0), captured at Day 21 and Day 7 post tamoxifen induction, respectively. Arrowheads in (D) labeled TdTomato^{+} cell bundle. (E) Quantitative analysis on TdTomato^{+} supporting cells, immature neurons, and mature neurons at Day 7 post tamoxifen induction, lineage-traced at P0 in the OE. (F) Quantitative analysis on TdTomato^{+} cells in the uninjured and injured OE at Day 7 and in the injured OE at Day 90 post lineage tracing, and on TdTomato^{+}/Sox2^{+} and TdTomato^{+}/OMP^{+} cells in the injured OE at Day 21 post lineage tracing. Immunostaining against GFP/Sox2, Tuj1 or OMP in the OE of LT mice at Day 21, lineage-traced at 3-month-old age (G, J, M) or Day 7 after tamoxifen induction, traced at P0 (H, K, N). (I, L) Confocal images of TdTomato^{+}/OMP^{+} cells in the OE lineage-traced at P0, captured at Day 31 post tamoxifen induction. (O) Schematic view of lineage tracing of Lgr5^{+} cells in methimazole-induced injured OE. Confocal images of TdTomato^{+} (P-R, P''-R'') or Lgr5-GFP^{+} cells (P'-R') in the injured and uninjured OE at Day 7 after lineage tracing. (S-U) Confocal images of TdTomato^{+} and Sox2^{+}, OMP^{+} or ICAM1^{+} cells in the injured OE at Day 21 post lineage tracing. (S'-U') were images of anti-GFP staining. Scale bars in (C, D, P-R) were 100 µm, and in (G, M, P'', S) were 25 µm.

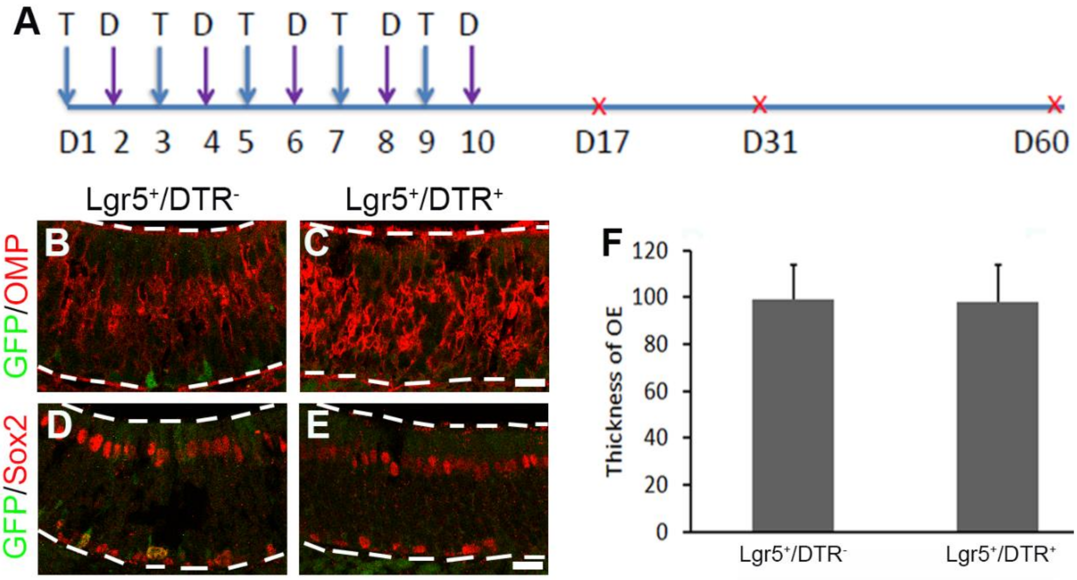

Supplementary Figure 5. Lgr5^{+} cells are not necessary in the OE homeostasis. (A) Scheme showing injection of tamoxifen (abbreviated as T) and diphtheria toxin (abbreviated as D) in Lgr5-EGFP-Cre^{ERT2}/Rosa-fl-STOP-fl-DTR (Lgr5^{+}/DTR^{+}) and Lgr5^{+}/DTR^{-} mice at 3-month-old age. (B, C) Immunostaining against GFP and OMP in the OE of Lgr5^{+}/DTR^{-} and Lgr5^{+}/DTR^{+} mice at Day 31 after tamoxifen injection. (D, E) Immunostaining against GFP and Sox2 at Day 31. (F) Quantitative analysis on OE thickness in Lgr5^{+}/DTR^{-} and Lgr5^{+}/DTR^{+} mice at Day 60 after tamoxifen injection. Dashed lines showed the apical and basal edges of the OE. Statistical significance was determined by unpaired t test. Scale bars were 25 μm.

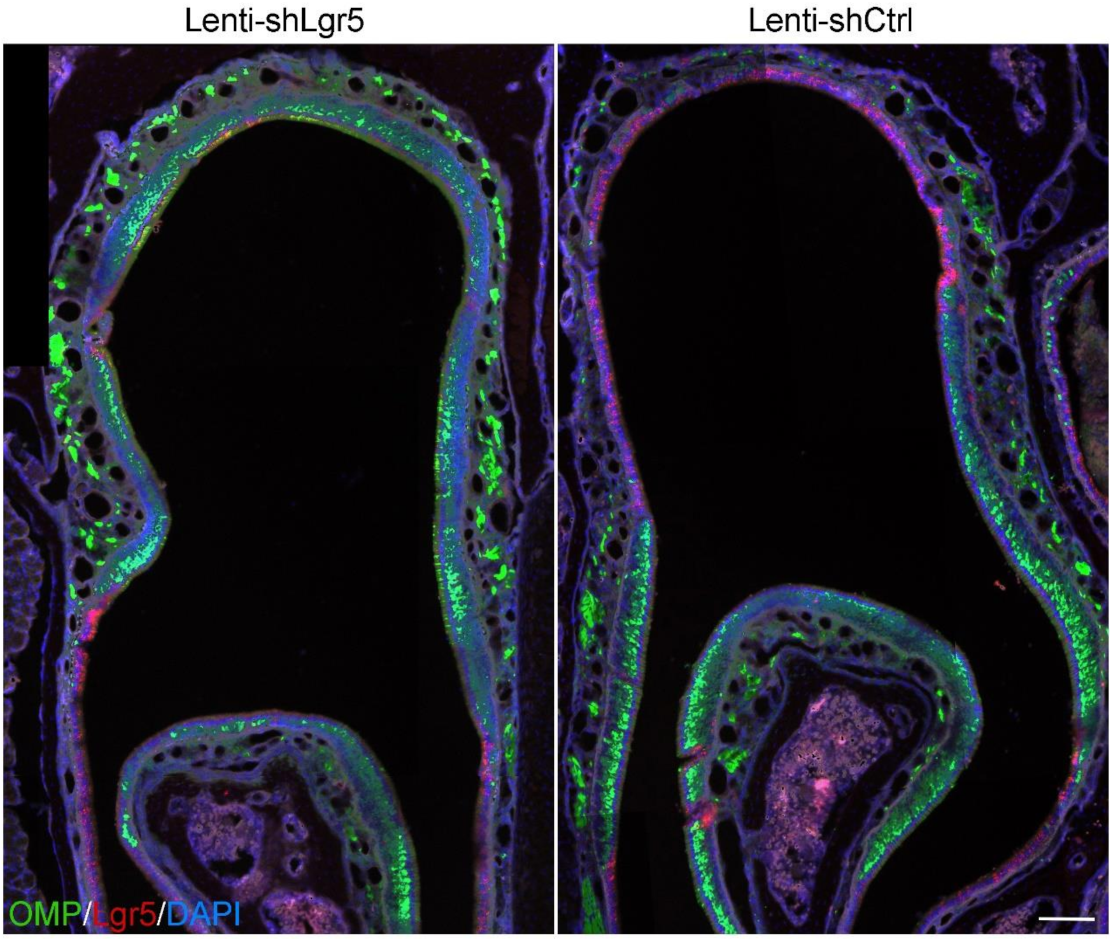

Supplementary Figure 6. Confocal images of OMP^{+} and Lgr5-mRNA^{+} signals in the OE of wide type C57BL/6J mice at Day 28 post injury, infected with Lenti-shLgr5 or Lenti-shCtrl. Scale bar, 100 μm.

Table S1. Primary antibodies used in this study

Primary antibody	Source/Vendor/Catalog no.	Cell type
Gt@Sox2	Santa Cruz Biotechnology, #sc-17320	Supporting cell, basal cell
Gt@Sox2	R&D Systems, # AF-2018	Supporting cell, basal cell
Rb@p63	Abcam, #ab63881	Horizontal basal cell
Gt@IL33	R&D Systems, #AF-3626	Supporting cell
Gt@DCX	Santa Cruz Biotechnology, # sc-8066	Immature olfactory sensory neuron
Rb@Krt14	Proteintech, #10143-1-AP	Horizontal basal cell
Rb@OMP	Abcam, # ab183947	Mature olfactory sensory neuron
Mo@Tuj1	Abcam, #ab78078	Immature olfactory sensory neuron
Gt@ICAM1	R&D Systems, #AF796	Horizontal basal cell
Chk@OMP	from Dr. Qizhi Gong(Chen et al., 2005)	Olfactory sensory neuron
Gt@NeuroD1	Santa Cruz Biotechnology, #sc-1086	Globose basal cell
Chk@GFP	Abcam, #ab13970	Lgr5-GFP$^+$ cell
Ra@GFP	ThermoFisher, #A11122	Lgr5-GFP$^+$ cell
Ra@PGP9.5	Proteintech, #14730-1-AP	Olfactory sensory neuron
Krt18	Abcam, # ab668	Supporting cell
